# Lip Flip Revisited: Clinical Outcomes and Neuromodulatory Basis of Upper Lip Eversion

**DOI:** 10.1007/s00266-026-05727-0

**Published:** 2026-03-31

**Authors:** Marcelo Germani, Victor R. M. Munoz-Lora, Pietra Roschel, Sebastian Cotofana

**Affiliations:** 1 Department of Biological Sciences, Bauru School of Dentistry, University of São Paulo, Bauru, Brazil; 2 Department of Facial Aesthetics, Guarulhos University, Guarulhos, São Paulo, Brazil; 3 HOF Pro Academy, Rio Verde, Goiás, Brazil; 4 Department of Biochemistry, Federal University of São Paulo, Paulista School of Medicine, São Paulo, Brazil; 5 Department of Plastic Surgery, Vanderbilt University Medical Center, Nashville, TN USA; 6 Centre for Cutaneous Research, Blizard Institute, Queen Mary University of London, London, United Kingdom; 7 Department of Plastic and Reconstructive Surgery, Guangdong Second Provincial General Hospital, Guangzhou, Guangdong Province China

**Keywords:** Lip augmentation, Botulinum toxin, Orbicularis oris muscle

## Abstract

**Abstract:**

The lip flip procedure, involving intramuscular injection of botulinum toxin into the orbicularis oris, has gained popularity as a minimally invasive alternative to lip fillers. In this prospective study, 17 women underwent standardized upper lip neuromodulation with 4U of onabotulinumtoxinA. Objective evaluation by stereophotogrammetry demonstrated a modest but significant increase in upper lip height at 15 days (p=0.011), with no change in lip volume. Patient-reported outcomes showed significant improvement in perioral appraisal scores (p<0.001) and high satisfaction (FACE-Q mean = 79.2 ±19.9; GAIS median = 1). All adverse events were mild and transient, including temporary numbness and functional limitation, resolving spontaneously within 30 days. These findings confirm that lip flip enhances lip projection through selective neuromodulation, not volumetric augmentation.

**Level of Evi„dence IV:**

Level of Evidence IV This journal requires that authors assign a level of evidence to each article. For a full description of these Evidence-Based Medicine ratings, please refer to the Table of Contents or the online Instructions to Authors www.springer.com/00266.

## Introduction

Lip augmentation remains one of the most demanded aesthetic procedures worldwide [1]. While hyaluronic acid (HA) fillers provide volumetric enhancement, the “lip flip” has emerged as a non-volumizing alternative, relying on botulinum toxin to relax the superficial orbicularis oris fibers and induce outward rolling of the upper lip [2].

Despite its increasing use, literature is limited to case reports and small series, with little objective evidence [3, 4]. This study evaluates the morphofunctional effects, safety, and patient satisfaction of the lip flip procedure using 3D stereophotogrammetry and validated patient-reported measures.

### Technique Description

**Fig. 1:**
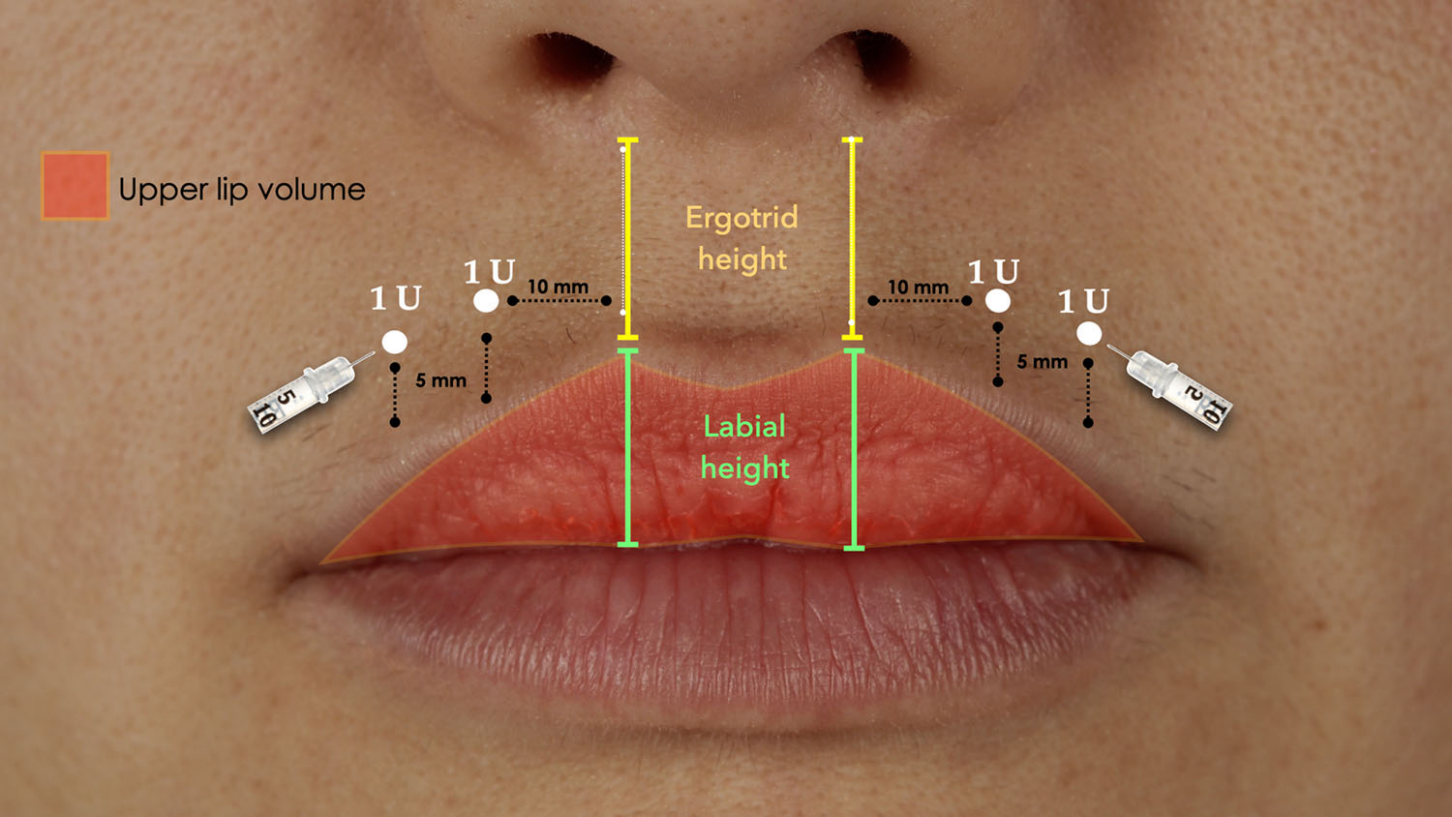
Standardized injection points and outcome measurement areas. White dots indicate the standardized injection points for neuromodulator application. Objective assessments included upper lip volume (highlighted in orange) and linear measurements of upper lip height and perioral height (green and yellow lines, respectively)

**Fig. 2:**
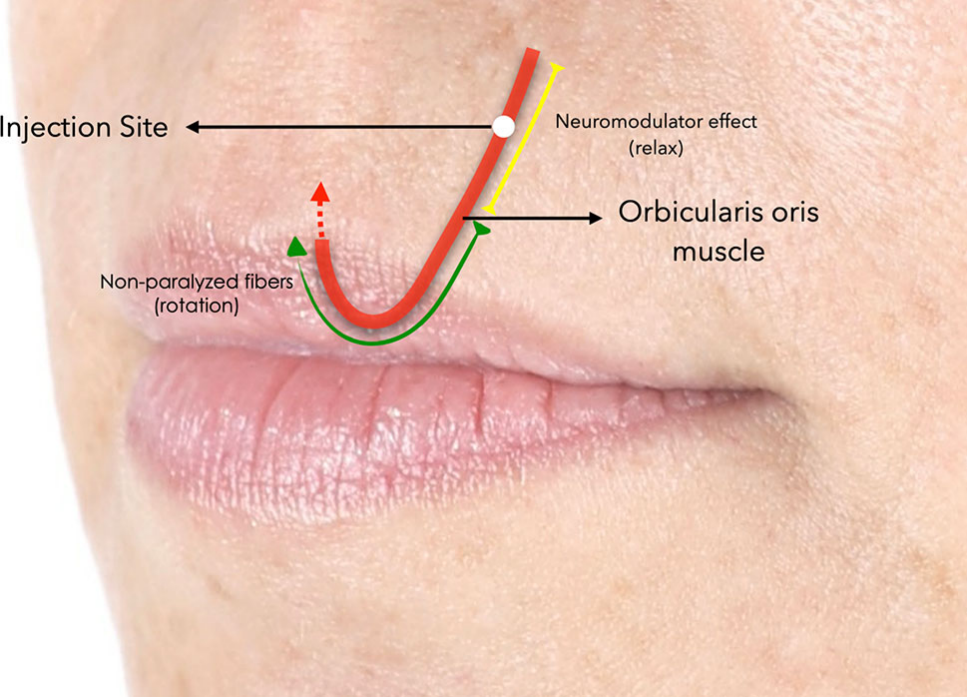
Schematic representation of the proposed biomechanical mechanism following neuromodulator application for upper lip eversion. The white dot indicates the injection site, targeting the orbicularis oris muscle. Relaxation occurs in the treated fibers (yellow line), while non-paralyzed fibers (green line) promote upward rotation of the upper lip vermilion, contributing to the lip eversion effect

All patients received treatment with onabotulinumtoxinA (Botox®, Abbvie, Dublin, Ireland), reconstituted at 1 mL/100 U. A total of 4 units were injected per patient, with 2 units per side (Fig. 1). Injections were placed with a 31G 8 mm insulin syringe, perpendicular to the skin at a depth of 2–3 mm, targeting the orbicularis oris muscle. The first point was located 5 mm above the vermilion border and 10 mm lateral to the philtrum, while the second was positioned 10 mm lateral to the first point, and also 5 mm above the vermilion border. This standardized placement targeted the superficial muscle fibers, aiming to reduce inward muscular tension and allow outward rolling of the upper lip (Fig. 2).

### Clinical Outcomes

**Fig. 3:**
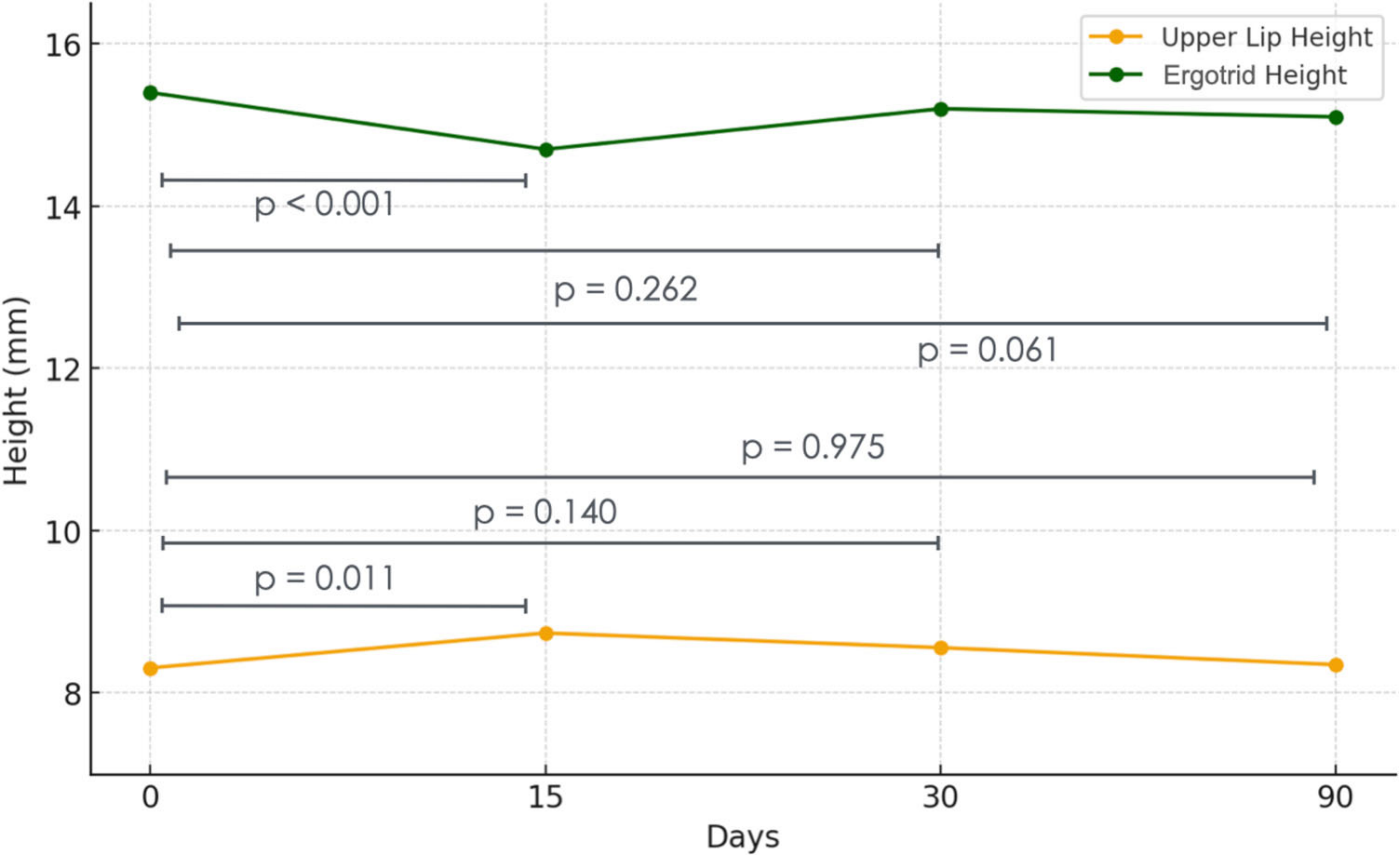
Line graph showing the mean values of upper lip and ergotrid height measured at baseline, 15, 30, and 90 days post-treatment. Both parameters demonstrated temporal fluctuations, with statistically significant changes over time detected by repeated measures ANOVA (p = 0.028 for upper lip height; p = 0.002 for perioral height)

**Fig. 4:**
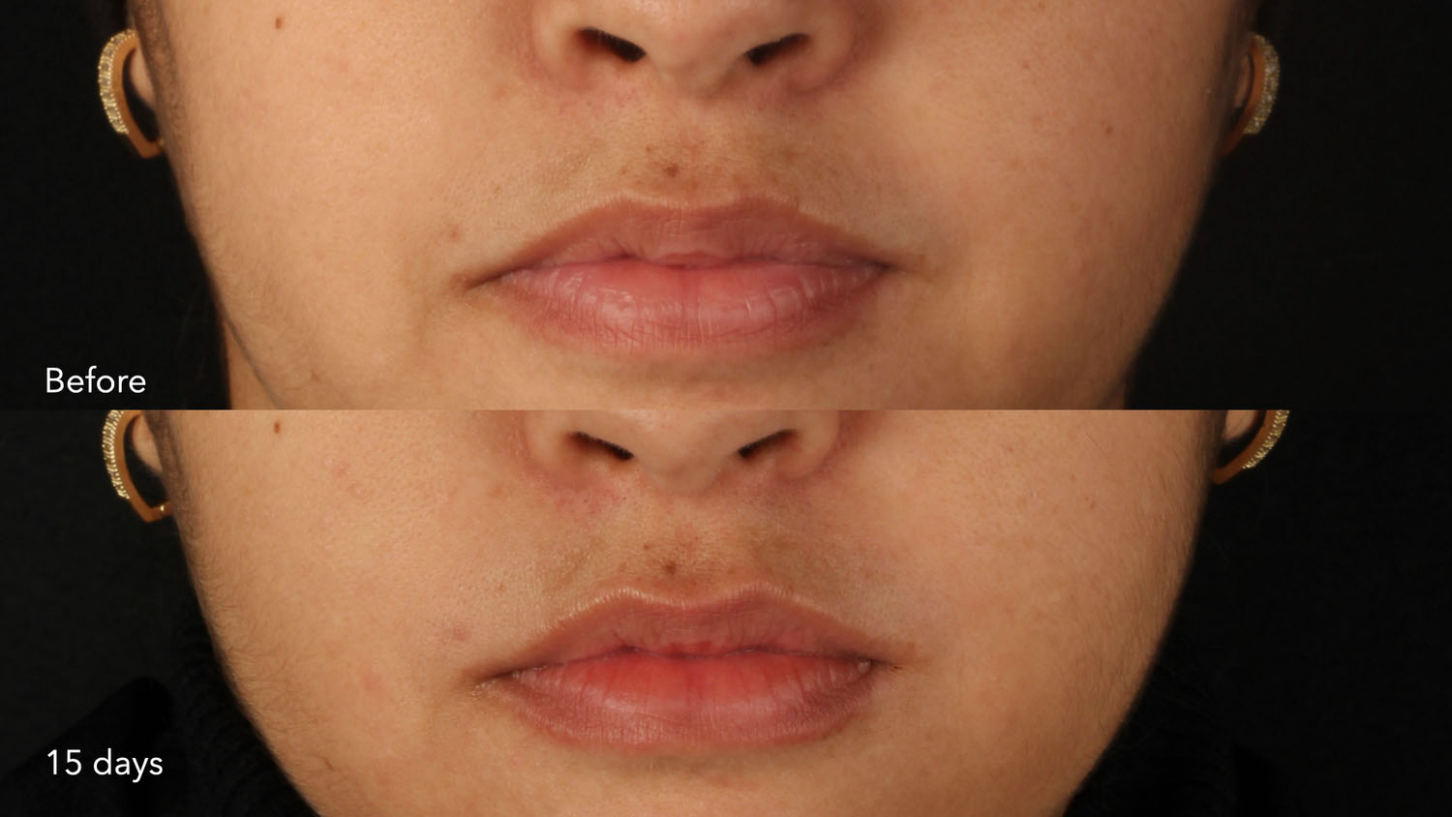
Assessment of a 31-year-old female patient (BMI = 26.35) at baseline and 15 days after treatment. Clinical evaluation focused on upper lip eversion following neuromodulator application

Seventeen women (mean age 40.4 ±11.3 years; mean BMI 25.4 ±2.9 kg/m^2^) were included. Three-dimensional stereophotogrammetry demonstrated that the mean upper lip height increased significantly from 8.31 mm (±1.03) at baseline to 8.74 mm (±1.07) at 15 days (p=0.011), before returning toward baseline at 30 days (8.56 mm; p=0.140) and 90 days (8.35 mm; p=0.975). Ergotrid height significantly decreased from 15.4 mm (±1.81) to 14.7 mm (±2.00) at 15 days (p<0.001), and then remained close to baseline at 30 (p=0.262) and 90 days (p=0.061) (Fig. 3). Lip volume showed no significant differences at any time point compared to baseline (p=0.152 at 15 days, p=0.127 at 30 days, and p=0.332 at 90 days). Representative standardized clinical images illustrate the subtle eversion of the upper lip observed after treatment, particularly at 15 days, despite the absence of volumetric augmentation (Fig. 4). These findings are consistent with the objective stereophotogrammetric data. Patient-reported outcomes confirmed clinical relevance: FACE-Q perioral appraisal scores significantly improved (39.2 ±10.5 to 33.8 ±10.7; p<0.001), FACE-Q Satisfaction with the result averaged 79.2 ±19.9 (p<0.001 vs neutral reference), and GAIS median was 1 (IQR 2.0; p<0.001). Safety assessment revealed that all participants experienced at least one mild, expected, and transient adverse event, including upper lip numbness (n=9), oral numbness (n=3), localized pain (n=2), and general discomfort (n=3). All resolved within 30 days and required no intervention.

## Discussion

This study suggests that the lip flip enhances upper lip projection primarily by neuromuscular modulation rather than volumetric augmentation. Selective weakening of the superficial fibers of the orbicularis oris muscle reduces inward muscular tension at the vermilion border, allowing subtle outward rotation of the upper lip without volumetric tissue expansion [5]. Objective 3D data confirmed a temporary increase in upper lip height without measurable volume gain, challenging the common misconception that the lip flip provides filler-like volumization [2]. High satisfaction despite the absence of volumetric change underscores the importance of patient perception and expectation management [6, 7]. Although the visual changes are subtle, they were consistently perceived by patients and reflected in improved patient-reported outcome measures, reinforcing the clinical relevance of the observed morphofunctional effects. The transient nature of the effect aligns with botulinum toxin pharmacodynamics and a possible compensatory muscle recruitment over time [8]. Clinically, the lip flip is most suitable for patients seeking subtle lip enhancement without additional volume (*i.e.,* particularly indicated for patients with adequate vermilion show at rest) and may serve as an entry-level or complementary procedure in lip aesthetics (Fig. 4). This treatment should be avoided in individuals with significant perioral muscle weakness or those with high expectations of volumetric lip enhancement. Adequate counseling on temporary functional effects is essential to ensure satisfaction.

This study has limitations inherent to its design, including a small sample size and lack of a comparator group. Dynamic functional assessments, such as motion imaging or smiling views, were not performed, and therefore, the findings should be interpreted as exploratory and mechanistic rather than evidence of comparative efficacy.

## Conclusion

Botulinum toxin into orbicularis oris provides temporary upper lip projection by selective neuromodulation, not by increasing volume. It is a safe, well-tolerated procedure associated with high patient satisfaction and only mild, transient adverse effects.
